# Parafoveal Processing and Transposed‐Letter Effects in Developmental Dyslexic Reading

**DOI:** 10.1002/dys.1791

**Published:** 2024-12-03

**Authors:** Julie A. Kirkby, Rhiannon S. Barrington, Denis Drieghe, Simon P. Liversedge

**Affiliations:** ^1^ Department of Psychology Bournemouth University Poole UK; ^2^ School of Psychology University of Southampton Southampton UK; ^3^ School of Psychology and Computing University of Central Lancashire Preston UK

**Keywords:** developmental dyslexia, eye movements, parafoveal processing, reading fluency, transposed‐letter effects

## Abstract

During reading, adults and children independently parafoveally encode letter identity and letter position information using a flexible letter position encoding mechanism. The current study examined parafoveal encoding of letter position and letter identity for dyslexic children. Eye movements were recorded during a boundary‐change paradigm. Parafoveal previews were either an identity preview (e.g., nearly), a transposed‐letter preview (e.g., enarly) or a substituted‐letter preview (e.g., acarly). Dyslexic readers showed a preview benefit for identity previews, indicating that orthographic information was encoded parafoveally. Furthermore, dyslexic readers benefitted from transposed‐letter previews more than substituted‐letters previews, demonstrating that letter identity was encoded independently to letter position during parafoveal processing. Although a transposed‐letter effect was found for dyslexic readers, they demonstrated a reduced sensitivity to detect transposed‐letters in later measures of reading, that is, go‐past times, relative to that found for typically developing readers. We conclude that dyslexic readers, with less rich and fully specified lexical representations, have a reduced sensitivity to transpositions of the first two letters of the upcoming word in preview. These findings are compatible with the view that orthographic representations of dyslexic children are not sufficiently specified.

## 1 Introduction

While several studies have reported disordered letter identification and letter‐order processing during reading in children with dyslexia (e.g., Friedmann, Dotan, and Rahamim 2010; Friedmann, Kerbel, and Shvimer 2010; Grainger et al. 2003; Lachmann and Van Leeuwen 2007; Pernet et al. 2006; Vidyasagar and Pammer 2010), there is a sparsity of studies focusing on letter identity and letter‐order during parafoveal processing in this population. The purpose of the current study was to examine letter identity and position information encoding parafoveally during sentence reading in children with and without dyslexia. Specifically, we included a group of children previously diagnosed with dyslexia, and two matched control groups: one matched to the dyslexic group for chronological age and another matched for reading age. By comparing dyslexic readers to two typically developing reading groups we can determine if deficits in parafoveal processing are specific to dyslexia, or due to a developmental lag indicative of their younger reading age.

Reading relies upon readers correctly and rapidly identifying phonological information from orthographic form. Thus, readers need to be able to sufficiently allocate their attention to identify the orthographic properties of a word, such as letter identity and letter position, and then determine the correct phonological mapping for that letter or combination of letters. Indeed, letter identity and letter position encoding are fundamental processes in visual word recognition that allow readers to distinguish the phonological output of anagrams such as *was* and *saw*.

While letter position is clearly an important aspect of visual word recognition, it is however, encoded with a level of flexibility. Studies of single word recognition in typically developing child readers (e.g., Acha and Perea 2008; Castles et al. 2007; Kohnen and Castles 2013; Lété and Fayol 2013; Paterson et al. 2015; Perea and Estévez 2008) have found that nonwords with a transposition of two letters (e.g., *jugde*), where the nonword unit contains the same orthographic content as the base word, are recognised more similar to the base word (e.g., *judge*) than nonwords with two SLs (e.g., *jupte*). This is known as the transposed‐letter (TL) effect and indicates that words with TLs significantly activate the lexical representation of the base word more than words with substituted‐letters (SLs). Therefore, letter identity encoding is not specific to letter position, and letter identity and letter position are encoded independently resulting in flexible letter position encoding.

Although beginning readers rely upon a letter‐by‐letter reading technique (Ehri 2005, 2010), Castles et al. (2007) suggest that children have an increased flexible letter position encoding mechanism, then more skilled readers. Castles et al. proposed that because children are less skilled at reading, they have a comparatively reduced vocabulary and therefore, a smaller range of words within their orthographic lexicon. Thus, during word identification, fewer competing lexical entries exist, and words are activated more flexibly. As such younger children are more likely to identify a word from a less accurate overlap of orthographic information than adults, who have wide‐ranging lexical representations. The more words children acquire in their orthographic lexicon, the more fine‐tuned the representations need to become. This was supported experimentally in Perea and Estévez (2008) who tasked Spanish beginning (7 years old), intermediate (9 years old) and adult readers to read aloud words with TLs (e.g., *cholocate*, where the base word was *chocolate*). They demonstrated that beginning readers made more errors (i.e., read aloud the base word when presented with the TL nonword), compared with both intermediate and adult readers. Acha and Perea (2008) demonstrated a difference between child and adult readers in the magnitude of the TL effect during lexical identification. Using a masked priming lexical decision task with Spanish beginning (7 years old), intermediate (11 years old) and adult readers, they demonstrated that the base words (e.g., *animal*), were responded to faster when primed by TL nonwords (e.g., *aminal*), than when primed by SL nonwords (e.g., *arisal*). Although the effect occurred for all three age groups, the TL effect was increased for beginning readers compared with both intermediate and adults.

These studies demonstrate a flexible letter position encoding mechanism, which has challenged traditional models of visual word recognition. According to models such as the Interactive Activation model (McClelland and Rumelhart 1981); the Dual Route Cascaded model (Coltheart et al. 2001); the Multiple Read Out model (Grainger and Jacobs 1996); the Activation‐Verification model (Paap et al. 1982); and the Parallel Distributed Processing model (Harm and Seidenberg 1999); a transposition (where two letters change positions) should be just as disruptive as a double letter substitution.

Models such as the SOLAR model (Davis 1999, 2010); the Open Bigram model (Grainger and Van Heuven 2003; Grainger, Kiyonaga, and Holcomb 2006); the Overlap model (Gómez, Ratcliff, and Perea 2008); and the SERIOL model (Whitney 2001), incorporate flexible mechanisms to encode letter position information and can account for the independent encoding of letter identity and position information. The Overlap model (Gómez, Ratcliff, and Perea 2008) assumes that when the nonword is presented briefly, the position that corresponds to each letter in the sequence is not precisely encoded; as a result, the visual information which corresponds to each letter is distributed over the entire word space (position uncertainty assumption). Other models, such as the Open Bigram (Grainger and Van Heuven 2003; see also Grainger, Kiyonaga, and Holcomb 2006) and the SERIOL model (Whitney 2001) assume that letter position is encoded through contextual information.

Previous research has shown that during reading, readers pre‐process an upcoming word (*n* + 1), while continuing to fixate the current word (*n*) in a sentence (reviewed in Rayner 1998, 2009; Schotter, Angele, and Rayner 2012; White et al. 2008). While recent models of word recognition (e.g., Davis 1999, 2010; Gómez, Ratcliff, and Perea 2008; Grainger and Van Heuven 2003; Whitney 2001) are useful in explaining flexible letter position encoding foveally, they have yet to be extended to consider flexible letter position encoding during parafoveal pre‐processing. They do, however, posit that both orthography and phonology contribute to lexical identification (e.g., Coltheart et al. 2001; Grainger and Ziegler 2011; Perry, Ziegler, and Zorzi 2007) and provide insight into pre‐processing of the upcoming word (*n* + 1). Grainger and Ziegler (2011) proposed that both orthographic and phonological characteristics of lexical stimuli exert an influence in lexical identification via two processing routes: a course‐grained processing route and a fine‐grained processing route. The coarse‐grained route permits direct semantic access from orthographic form and allows a level of flexibility in orthographic encoding. The fine‐grained route allows access to semantics via commonly co‐occurring letter patterns being processed and mapped onto their corresponding phonological representations with less encoding flexibility. Skilled readers show a decreased reliance on phonological decoding (serial sounding out of letter sounds) and an increased reliance on coarse‐grained processing. However, the fine‐grained processing still occurs and allows phonological recoding. There is evidence for fine‐grained processing in adult, teen readers, and children as young as 8 years old (Blythe et al. 2018, 2020; Milledge, Blythe, and Liversedge 2021; Pollatsek et al. 1992), where skilled readers have been shown to pre‐process phonology from word (*n* + 1; phonological recoding) and, importantly, this is modulated by orthographic similarity.

Pagán, Blythe, and Liversedge (2016) used the boundary‐change paradigm (Rayner 1975) to explore the TL effect during parafoveal processing for typically developing children—with a mean age of 9 years old. (see also Johnson, Oehrlein, and Roche 2018; Marx et al. 2016; Sperlich, Schad, and Laubrock 2015; Vorstius, Radach, and Lonigan 2014). Pagán, Blythe, and Liversedge (2016) demonstrated that TL effects occur in the initial trigram of the parafoveal word—in the first two letters (letter positions 1 and 2; *acptain*‐*imptain*, base word *captain*) and for the second two letters (letter positions 2 and 3; *cpatain‐cgotain*) but not for non‐adjacent letters (i.e., letters 1 and 3; *pactain‐gartain*).

The intrinsic importance of the initial letters (e.g., Johnson and Eisler 2012; Lima and Inhoff 1985) may be related to the need for sequential activation of phonological codes, particularly in determining the phonological onset of the word and activation of the phonological representations—where phonological information is encoded before lexical access (for a review see Leinenger 2014). While orthographic codes are activated earlier than phonological codes (Lee, Rayner, and Pollatsek 1999), phonological information clearly still impacts lexical activation. Milledge, Blythe, and Liversedge (2021) suggested that the first letters of an upcoming word in preview are critical to phonological pre‐processing, and that orthographic similarity of preview and target word was important, particularly for children compared with skilled readers.

While there has been progress in research on the development of parafoveal processing in typically developing child readers (e.g., Häikiö et al. 2009; Häikiö, Bertram, and Hyönä 2010; Johnson, Oehrlein, and Roche 2018; Pagán, Blythe, and Liversedge 2016; Marx et al. 2015, 2016; Tiffin‐Richards and Schroeder 2015), there is still a paucity of research exploring parafoveal processing in dyslexic child readers, particularly during sentence reading. There are, however, studies that have found evidence for parafoveal processing for dyslexic readers during Rapid Automised Naming (RAN; Wolf and Denckla 2005) in children with dyslexia (Yan et al. 2013).

Yan et al. (2013) explored parafoveal processing for Chinese dyslexic children, during the RAN task (Wolf and Denckla 2005). They found the dyslexic children were less efficient at processing orthographic parafoveal information compared with chronological age matched typically developing children. These data were recorded during two RAN tasks; (1) a continuous RAN task (in which all letters were presented at the same time) and (2) a discrete RAN task (where one letter was presented at a time, therefore removing the possibility of parafoveally processing the next letter). Through the inclusion of these two variations of the RAN task, Yan et al. showed that by removing the upcoming letter (i.e., in the discrete condition) both groups of readers were disrupted in terms of their naming times and viewing durations. However, children with dyslexia were less disrupted by the discrete RAN condition relative to the typically developing child readers. Consequently, while children with dyslexia can use parafoveal information, Yan et al. demonstrated that they were less efficient in parafoveal processing of the upcoming letter (*n* + 1) compared with that found in typically developing children of the same age. Yan et al. concluded that due to greater attentional resources required for foveal processing, parafoveal processing for dyslexic child readers was limited.

A large literature examining effects of foveal load (e.g., Henderson and Ferreira 1990) has suggested that increased foveal load reduces the amount of parafoveal processing on word *n* + 1 conducted prior to a saccade from word *n* in skilled adult reading. Silva et al. (2016) found a reduced parafoveal preview benefit for adult dyslexics during a naming task. They concluded that reduced parafoveal preview benefit is a core deficit in dyslexia, which they suggest is a direct consequence of dyslexic reading deficits. As such, during reading compared with naming tasks, dyslexic readers may experience a reduction in parafoveal preview benefit as lexical processing consumes significant cognitive resources, which might represent an increased foveal load. Kirkby et al. (2022) found that adult dyslexic readers were less able to benefit from correct letter identity information (i.e., in the letter transposition previews), which they suggest was due to a lack of direct mapping of orthography to phonology. However, whether this might be a consequence of increased foveal load for dyslexic compared with skilled readers was not discussed.

The decision to manipulate the transposition and substitution of letters specifically in the initial two letters of the parafoveal word is grounded in empirical research. Pagán, Blythe, and Liversedge (2016) found that TL effects occur prominently in children around 9 years old for the first two letters of a parafoveal word. This suggests that the initial letters play a crucial role in early word recognition processes. Furthermore, Milledge, Liversedge, and Blythe (2022) identified the first letter of a word in preview as particularly significant in English reading. Their study indicates that the first letter is vital for orthographic encoding, which is essential for the efficient early processing of phonology. Thus, the manipulation of the first two letters in the parafoveal word in the current experiment is aimed at leveraging these findings to explore their impact on readers with dyslexia.

Using the boundary‐change paradigm with identity previews (IP; e.g., *nearly*), TL previews (e.g., *enarly*) and SL previews (e.g., *acarly*) the current research aimed to clarify whether children with dyslexia exhibit parafoveal preview benefit during silent sentence reading and the extent to which the TL effect is evident in dyslexic reading compared with typically developing child readers matched for chronological age or reading age.

Based on the findings of Pagán, Blythe, and Liversedge (2016), Tiffin‐Richards and Schroeder (2015) and Yan et al. (2013) it was predicted that typically developing readers would have the shortest reading times following an IP compared with a non‐identical preview. Where letter identity and position information would be extracted from the parafovea. Also, based on the results of Pagán, Blythe, and Liversedge (2016), it was predicted that a TL effect where subsequent reading times would be shorter following a TL preview compared with a SL preview for typically developing children.

Further, in line with Yan et al. and the large literature examining the effect of foveal load (Henderson and Ferreira 1990), it was predicted that due to greater attentional resources required for foveal processing, parafoveal encoding of letter identity and letter position may be limited for dyslexic children. As such while it was predicted that dyslexic readers would benefit from IPs (similar to that found for adults with dyslexia, Kirkby et al. 2022), dyslexic readers may be less able—due to increased foveal load—to benefit from correct letter identity information when those letters were transposed (i.e., in the letter transposition previews) and, similar to adults with dyslexia, rely more on direct mapping of orthography to phonology in parafoveal processing.

In addition to testing these predictions for parafoveal processing, we fully documented global eye movement behaviour for dyslexic child readers compared with typically developing child readers. Any differences in eye movement behaviour between these three groups will further our knowledge of the condition and aid in documenting the difficulty experienced by dyslexic readers in linguistic information processing.

## 2 Method

### 2.1 Participants

Participants included 18 children with developmental dyslexia (mean age 10 years 4 months, reading age 8 years and 0 months), 28 typically developing children matched to the chronological age of the dyslexic readers (mean age 10 years 2 months, reading age 12 years and 7 months) and 28 typically developing children matched to the reading age of the dyslexic children (mean age 8 years 8 months, reading age 8 years and 3 months). Children with dyslexia had a prior, independent diagnosis of dyslexia, through their local education authority (supported by offline measures of reading ability and RAN speed reported in the results). All participants were native English speakers with normal or corrected to normal vision and were recruited from local schools in Bournemouth and Poole. The children performed within or above the normal range for IQ (IQ ≥ 90; Wechsler 1999). Two significant one‐way ANOVAs found (1) chronological age differed across groups, *F* (2,73) = 47.66, *p* < 0.001—the dyslexic group differed to the reading age matched group [*t* (71) = 8.05, *p* < 0.001] but there was no significant difference between the chronological‐age matched group and the dyslexic group [*t* (71) = 0.52 *p* = 1.00] and (2) the reading age differed across the three groups, *F* (2,73) = 48.08, *p* < 0.001—the dyslexic group differed to the chronological age matched group [*t* (71) = 8.09, *p* < 0.001] but there was no significant difference between the reading age matched group and the dyslexic group [*t* (71) = 0.53, *p* = 1.00].

### 2.2 Apparatus

Eye movements were recorded from the right eye using a SR Research Eyelink 1000 eye‐tracker. Sentences were presented at a viewing distance of 660 mm on a 21‐in. Formac ProNitron 21/750 monitor with a screen resolution of 1024 × 768 pixels and a refresh rate of 120 Hz. Sentences were presented in black 14pt. Courier New font on a white background. At this viewing distance, three characters equalled 1° of visual angle.

### 2.3 Design and Stimuli

Three parafoveal preview conditions were presented using the boundary‐change paradigm (Rayner 1975). Parafoveal previews of the target words were either (1) IP, (2) a TL nonword or (3) a SL nonword. The manipulation occurred in the initial two letters of the target word. For the TL conditions, the positions of the two initial letters were switched, and, for the SL conditions the initial two letters were replaced with visually similar letters (i.e., ascenders were replaced with ascenders and descenders with descenders). Target words were always six letter words and preceded by a five or six letter pre‐target word and presented at or around the middle of the sentence. All pre‐target and target words were high frequency. The mean frequency of the pre‐target word was 618 counts per million and the mean frequency of the target word was 409 counts per million. Frequency counts were taken from the Children's Printed Word Database (CPWD; Masterson et al. 2010) as this best reflects frequency counts for children. Sentences were single line sentences ranging from 9 to 12 words (45–60 characters). The stimuli consisted of 60 sentence frames. For each sentence frame there were three versions corresponding to the three parafoveal preview conditions. Three experimental lists were constructed whereby each list contained a different version of each sentence frame and the parafoveal preview manipulations were randomised across the three experimental lists, so each participant saw 20 sentences from each of the three preview conditions.

The eye contingent boundary‐change was located at the end of the pre‐target word and to the left of the space preceding the target word. When the eyes moved past the invisible boundary, the target word changed from the parafoveal preview to the target word. The correct target word then remained in the sentence throughout the remaining duration of the trial. Display changes were undetected by the child readers, occurring during a saccade when visual information is suppressed (Matin 1974).

### 2.4 Offline Measures of Reading Ability and IQ

All participants completed a range of offline tests. IQ was measured using two subtests of the Wechsler Abbreviated Scale of Intelligence (WASI; Wechsler 1999); (i) the vocabulary subtest, (ii) the matrix reasoning subtest. The word reading and pseudoword reading subtests of the *Wechsler Individual Achievement Test—Second Edition* (WIAT‐II; Wechsler 2005) provided standardised measures of reading ability based upon a child's chronological age. Thus, in the instance of dyslexic readers, they show a standardised score that represents their low reading skills relative to their chronological age. The result from the word reading subtest were used to determine each child's reading age, whereby the raw score taken from word reading subtest indicates at what age‐level a child is reading. As such, reading age was used as a comparative measure across all children regardless of their chronological age. All children completed the number and letters measures of the RAN (Wolf and Denckla 2005) task which provided further support for the comparisons between the dyslexic and reading age matched groups.

### 2.5 Procedure

Participants sat in front of a computer screen with their head positioned in a forehead restraint and chin rest to minimise head movements. They were instructed to read the sentences silently for comprehension and to press a button on a gamepad once they had finished reading. A three‐point calibration was conducted prior to the experimental trials; an accurate calibration was accepted when the average errors in the validation were below 0.3° of visual angle (equal to one character space). Calibrations were confirmed throughout the experiment and repeated when required. Each trial began with a gaze contingent box (a small black square) presented on the left‐hand side of the screen, positioned so that the initial letter of the sentence occupied the same location. Once the participant had fixated the square for 250 ms, the sentence appeared on the screen. Participants then read the sentence silently and terminated the trial with a button press. After 25% of the experimental sentences a “yes/no” comprehension question appeared; participants were required to press a corresponding button to answer the question.

### 2.6 Statistical Analysis

Prior to the analysis, fixations less than 80 ms were either merged into nearby longer fixations or excluded and fixations more than 800 ms were excluded from the data set (5.49% of fixations). Additional trials were excluded based upon the following criteria; (1) when the boundary was triggered prior to a saccade being made across the boundary, (2) when the display change completed more than 10 ms after a fixation landing on the target word, (3) when the end of a saccade briefly crossed the boundary but the successive fixation remained in a position before the boundary, (4) when participants blinked on either the pre‐target or target word, (5) when the participants skipped either the pre‐target or target word (an additional 5.3%). In total 1522 trials were removed from the analyses (34% of the dataset), data were excluded similarly across groups and conditions.

## 3 Results

Analyses were conducted for both global and local measures. Global measures refer to results from all the fixations within the sentence whereas local measures were based solely on the eye movements that occurred on the target word. Data were analysed using linear mixed models (LMMs) using the lme4 package (version 1.1‐20) in R (version 3.5.2). For global analyses, reading group was the fixed factor for all models. For local analyses, both reading group and preview condition were fixed factors for all models. Participants and items were specified as random effects for both global and local analyses. For each dependent measure, a “full” random structure was implemented including all varying intercepts and slopes of the main effects and their interaction (maximal random effects structure as suggested by Barr et al. 2013). If the “full” model failed to converge, or there were too many parameters to fit the data (as indicated by nearly perfect correlations of 0.99, 1, −0.99 or − 1 in the random structure), the random structure was systematically trimmed (first by removing correlations between random effects, and if necessary, also by removing their interactions). Contrasts for the global analyses were used for reading group with the first contrast exploring chronological age matched children compared with dyslexic and reading age matched children (+1, −0.5, −0.5), and the second contrast exploring dyslexic children compared with reading age matched children (−1, +1). Given our specific predictions for the previews, successive difference contrasts were used for preview condition (comparing identical previews and TL previews [−1/+1], followed by TL previews and SL previews [−1/+1]). For each contrast we report beta values (*b*), standard error (SE) and *t* or *z* statistics. Fixation time analyses were carried out on log‐transformed models to increase normality and count data were analysed using generalised LMMs following a poisson distribution (GLMMs).

### 3.1 Eye Tracking Comprehension Questions

The mean accuracy in comprehension score was 87.78% correct for dyslexic children, 91.43% for chronological age matched children and 88.57% for reading age matched children. There were no significant differences between the comprehension scores for the three groups, *F* (2,71) = 1.34, *p* = 0.27, suggesting all reading groups were able to read these sentences well enough to correctly respond to the comprehension questions.

### 3.2 Offline Measures of Reading Ability and IQ

Mean scores for all offline tests are presented in Table 1. ANOVAs were conducted to explore the differences between reading groups across the offline measures. There was a significant main effect for word reading efficiency (*F* (2,71), 29.70, *p* < 0.001) and pseudoword reading (*F* (2,71), 19.40, *p* < 0.001). As both measures are presented as standardised scores based upon chronological age, dyslexic children performed significantly lower compared with both groups of typically developing children—dyslexic compared with reading age matched group for word reading efficiency [*t* (71) 5.32, *p* < 0.001] and pseudoword reading [*t* (71) = 4.07, *p* < 0.001] and dyslexic compared with chronological age matched group for word reading efficiency [*t* (71) 6.99, *p* < 0.001] and pseudoword reading [*t* (71) = 4.07, *p* < 0.001]. Although there were no significant differences between the three reading groups for the letter subset of the RAN (*F* (2,71), 1.52, *p* = 0.225), there was a significant effect for the number subset of the RAN (*F* (2,71), 5.80, *p* = 0.005); children with dyslexia performed significantly slower than the age matched children [*t* (71) 2.90, *p < 0*.01] and reading age matched children [*t* (71) 3.18, *p* = 0.01].

**TABLE 1:** Mean scores for the offline tests and age data for children with dyslexia, reading age matched children and chronological age matched children.

	Dyslexic children	Reading age matched group	Chronological age matched group
Chronological age	10 years 4 months	8 years 8 months	10 years 2 months
Reading age	8 years 0 months	8 years 3 months	12 years 7 months
IQ	101 (10)	111 (14)	113 (10)
Word reading	84.33 (9.65)	102.54 (11.46)	108.25 (12.12)
Pseudoword reading	88.17 (10.30)	103.54 (14)	108.57 (12.16)
RAN letters	94.54 (12.12)	99.89 (15.95)	101.72 (12.54)
RAN numbers	95.00 (10.68)	106.56 (11.69)	105.28 (12.35)

*Note:* Standard scores are provided for IQ, word reading, pseudoword reading and RAN numbers and letters. Standard deviations are shown in parentheses.

### 3.3 Global Measures

The following global measures were included in the analyses; total sentence reading time, average saccade amplitude, average forward and regressive fixation duration and total number of forward and regressive fixations per sentence (see Table 2 for means and SDs; Tables 3 and 4 for model outputs).

**TABLE 2:** Average global reading measures: total reading time (ms), saccade amplitude (degrees), average fixation duration (ms) and number of fixations, for children with dyslexia, typically developing children matched for reading age and typically developing children matched for chronological age.

					Fixation duration				Fixation count			
	Total time		Saccade amplitude		Forward		Regressive		Forward		Regressive	
	M	SD	M	SD	M	SD	M	SD	M	SD	M	SD
Dyslexic readers	7120	3171	1.93	0.59	288	46	288	81	13.06	4.29	4.87	2.93
Reading age matched	6350	2883	2.01	0.62	282	50	268	89	12.75	4.23	4.25	2.78
Chronological age matched	4374	1843	2.26	0.57	245	40	225	111	10.79	3.32	3.4	2.79

**TABLE 3:** Model output for LMMs conducted for global reading measures of total reading time (ms), saccade amplitude (degrees), average forward fixation duration (ms) and average regressive fixation duration.

	Total time				Average saccade amplitude				Forward fixation duration				Regressive fixation duration			
	*b*	SE	*t*	*p*	*b*	SE	*T*	*p*	*b*	SE	*t*	*p*	*b*	SE	*t*	*p*
Intercept	8.61	0.04	**229.52**	**< 0.001**	0.67	0.02	**29.5**	**< 0.001**	5.59	0.01	**397.8**	**< 0.001**	5.57	0.01	**433.39**	**< 0.001**
DR + RA versus CA	−0.43	0.07	**−5.88**	**< 0.001**	0.16	0.05	**3.57**	**< 0.001**	−1.61	0.03	**−5.67**	**< 0.001**	−0.15	0.03	**−5.72**	**< 0.001**
DR versus RA	0.16	0.09	1.78	0.1	−0.08	0.06	−1.44	1	0.02	0.04	0.6	1	0.06	0.03	**1.97**	0.1

*Note:* Significant *t* values (≥ 1.96 of standard error) are marked in bold.

**TABLE 4:** Model output for GLMMs conducted for global reading measures of forward fixation count and regressive fixation count.

	Forward fixation count				Regressive fixation count			
	*b*	SE	*z*	*p*	*b*	SE	*z*	*p*
Intercept	2.49	0.03	**98.8**	**< 0.001**	1.34	0.06	**24.39**	**< 0.001**
DR + RA versus CA	−0.19	0.05	**−3.88**	**< 0.001**	−0.37	0.11	**3.41**	**< 0.001**
DR vs. RA	0.06	0.06	1.03	1	0.17	0.13	1.25	1

*Note:* Significant *z* values (≥ 1.96 of standard error) are marked in bold.

#### 3.3.1 Total Reading Time

Dyslexic readers and reading age matched children had significantly longer total reading times compared with those found for the chronological age matched children. Thus, demonstrating increased difficulty with linguistic processing relative to the more skilled child readers. There was also a trend in the total reading time data to suggest that children with dyslexia had longer durations than the reading age matched children (*t* = 1.78, *p* = 0.1) although this was non‐significant.

#### 3.3.2 Saccade Amplitude

Dyslexic readers and reading age matched children had shorter saccade amplitudes compared with those of the more skilled child readers. No significant differences in saccade amplitudes were found between dyslexic children and reading age matched children.

#### 3.3.3 Forward and Regressive Fixation Durations

For both forward and regressive fixation durations, dyslexic readers and younger typically developing children, matched on reading‐age made longer fixations compared with the fixation durations found for the older more skilled child readers, again indicative of increased linguistic processing difficulty. For forward fixation duration, there was no difference in the duration of fixations for the dyslexic children compared with the reading age matched children. For regressive fixation duration, however, there was a significant difference between the dyslexic children and those matched for reading‐age. Dyslexic readers made longer regressive fixations, indicating that even though these groups were matched on reading‐age, dyslexic readers still require longer regressive fixation durations then non‐dyslexic readers.

#### 3.3.4 Forward and Regressive Fixation Counts

Dyslexic readers and the group match on reading‐age made more forward and regressive fixations compared with that found for the group matched on chronological‐age. There were no significant differences between the number of fixations made for the dyslexic children and the number made by the younger typically developing reading‐age matched children, for either forward or regressive fixations.

### 3.4 Local Measures

The following measures were used to analyse the embedded target words; first fixation duration, single fixation duration, gaze duration, go‐past time, fixation count and landing position. Table 5 provides means and SD for these measures across reading group and preview condition. Table 6 provides the LMM outputs and Table 7 provides LMM outputs for the simple effects' analysis for an interaction between group and preview occurred.

**TABLE 5:** Average local reading measures: mean first fixation duration, single fixation duration, gaze duration, go‐past time, landing position and fixation count and for the target word, as a function of preview condition and reading group.

	Identical preview	Transposed	Substituted
First fixation duration (ms)			
Dyslexic	322 (138)	319 (132)	320 (131)
Reading age match	312 (128)	305 (134)	323 (137)
Chronological age match	252 (93)	270 (98)	276 (100)
Single fixation duration (ms)			
Dyslexic	352 (137)	341 (127)	371 (137)
Reading age match	325 (122)	329 (135)	358 (131)
Chronological age match	265 (96)	288 (103)	301 (105)
Gaze duration (ms)			
Dyslexic	515 (354)	579 (451)	588 (402)
Reading age match	455 (269)	497 (309)	486 (262)
Chronological age match	342 (189)	353 (154)	371 (162)
Go‐past time (ms)			
Dyslexic	753 (876)	741 (653)	807 (584)
Reading age match	634 (622)	700 (614)	655 (490)
Chronological age match	436 (379)	484 (424)	480 (438)
Landing position			
Dyslexic	2.84 (1.43)	2.90 (1.51)	2.84 (1.46)
Reading age match	3.08 (1.62)	3.05 (1.59)	3.01 (1.57)
Chronological age match	3.09 (1.62)	3.10 (1.55)	3.08 (1.52)
Fixation count			
Dyslexic	2.60 (1.84)	2.55 (1.76)	2.69 (1.87)
Reading age match	2.20 (1.33)	2.36 (1.45)	2.40 (1.47)
Chronological age match	1.90 (1.09)	1.95 (1.12)	1.94 (1.13)

*Note:* Standard deviations are shown in parentheses.

**TABLE 6:** Model output for LMMs conducted for local reading measures of first fixation duration (ms), single fixation duration (ms), gaze duration (ms), go‐past time (ms), landing position (characters) and fixation count.

	First fixation				Single fixation				Gaze duration			
	*b*	SE	*t*	*p*	*b*	SE	*t*	*p*	*b*	SE	*t*	*p*
Intercept	5.62	1.71	**329.32**	**< 0.001**	5.71	2.2	**257.14**	**< 0.001**	6	3.13	**191.83**	**< 0.001**
IP versus TL	1.05	1.68	0.36	= 1	2.39	2.08	1.15	= 1	7.22	1.97	**3.66**	**< 0.001**
TL versus SL	2.51	1.69	1.48	= 1	7.05	2.2	**3.21**	**< 0.01**	5.67	1.99	**2.85**	**< 0.01**
DR + RA versus CA	−1.62	3.38	**−4.78**	**< 0.001**	−1.94	4.24	**−4.58**	**< 0.001**	−0.39	5.89	**−5.75**	**< 0.001**
DR versus RA	2.92	4.24	0.69	= 1	2.46	5.45	0.45	= 1	1.6	7.37	**2.17**	**< 0.05**
IP versus TL: DR + RA versus CA	8.88	3.38	**2.63**	**< 0.05**	9.07	4.03	**2.25**	**< 0.05**	−1.87	3.97	−0.47	= 1
TL versus SL: DR + RA versus CA	−9.27	3.39	−0.27	= 1	−3.63	4.2	−0.86	= 1	−1.35	3.98	−0.03	= 1
IP versus TL: DR versus RA	1.49	4.31	0.35	= 1	−3.54	5.48	−0.65	= 1	−1.11	5.06	−0.22	= 1
TL versus SL: DR versus RA	−5.52	4.37	−1.26	= 1	−6.17	5.88	−0.11	= 1	6.71	5.13	1.31	= 1

	Go past time				Landing position				Fixation count			
	*b*	SE	*t*	*p*	*b*	SE	*t*	*p*	*b*	SE	*t*	*p*
Intercept	6.22	0.04	**174.21**	**< 0.001**	2.98	5.65	**52.56**	**< 0.001**	0.81	0.03	**25.51**	**< 0.001**
IP versus TL	0.09	0.03	**3.51**	**< 0.001**	1.15	6.99	0.16	= 1	0.02	0.03	**0.72**	= 1
TL versus SL	0.05	0.02	**2.33**	**< 0.05**	−3.7	7.06	−0.52	= 1	0.03	0.03	**1.02**	= 1
DR + RA versus CA	−0.4	0.07	**−6.13**	**< 0.001**	1.63	1.06	1.53	= 1	−0.25	0.06	**−4.12**	**< 0.001**
DR versus RA	0.18	0.08	**2.16**	**< 0.05**	−2.06	1.33	−1.55	= 1	0.15	0.07	**2.04**	**< 0.05**
IP versus TL: DR + RA versus CA	0.07	0.05	1.36	= 1	−5.06	1.41	−0.04	= 1	0.01	0.07	0.13	= 1
TL versus SL: DR + RA versus CA	−0.09	0.05	−1.73	**< 0.01**	3.4	1.41	0.02	= 1	−0.05	0.06	−0.82	= 1
IP versus TL: DR versus RA	−0.06	0.06	−0.97	= 1	9.35	1.79	0.53	= 1	−0.08	0.08	−1.01	= 1
TL versus SL: DR versus RA	0.13	0.06	**2.04**	**< 0.05**	−2.41	1.82	−0.13	= 1	0.05	0.08	0.61	= 1

*Note:* Significant t or *z* values (≥ 1.96 of standard error) are marked in bold.

**TABLE 7:** LMM output for simple effects analysis exploring transposed‐letter (TL) compared with substituted‐letters (SL) for dyslexic readers in go‐past time (ms).

	Go‐past time			
	*b*	SE	*t*	*p*
Intercept	6.40	0.09	**70.66**	**< 0.001**
TL versus SL	0.16	0.05	**3.19**	**< 0.01**

*Note:* Significant *t* values (≥ 1.96 of standard error) are marked in bold.

#### 3.4.1 First Fixation Duration

For first fixation duration there was a main effect of group; dyslexic readers and reading age matched younger group of typically developing children had longer first fixation durations than chronological age matched children. There were no significant differences in first fixation durations for children with dyslexia and typically developing children matched for reading age. The main effects for both preview condition contrasts (IPs compared with TL previews and TL previews compared with SL previews) were non‐significant but there was a significant interaction in which shorter first fixation durations were made on IPs than TL previews for the chronological age matched children only (see Table 6). Thus, the chronological age matched children showed a benefit of parafoveal information during reading in early reading measures, that is, first fixation duration. Whereas dyslexic readers and reading age matched children did not. All other interactions did not reach significance.

#### 3.4.2 Single Fixation Duration

In single fixation duration, there was a similar pattern to that of first fixation duration. Dyslexic children and reading age matched children made longer single fixation durations than chronological age matched children, again, indicative of less efficient linguistic processing compared with the chronological age matched children who had a higher reading age. There was no significant difference between the single fixation durations for children with dyslexia compared with those for the reading age matched children. The main effect comparing IPs with TL previews showed that there was no significant difference in single fixation duration between these conditions; however, there was an interaction in which shorter single fixation durations were made following an IP compared with following a TL preview specifically for the chronological age matched children (see Table 6). There was a significant main effect, whereby shorter single fixations were made for TL previews than SL previews. Where all reading groups demonstrated a TL effect. The additional interactions were not significant.

#### 3.4.3 Gaze Duration

Dyslexic children and reading age matched children required longer gaze durations compared with the older chronological age matched children. In addition, dyslexic children required longer gaze durations than the younger reading age matched children. For the preview condition contrasts, both main effects were significant; shorter gaze durations were made following an IP compared with a TL previews and following a TL preview compared with a SL preview (see Table 6). None of the interactions was significant.

#### 3.4.4 Go‐Past Time

**FIGURE 1:**
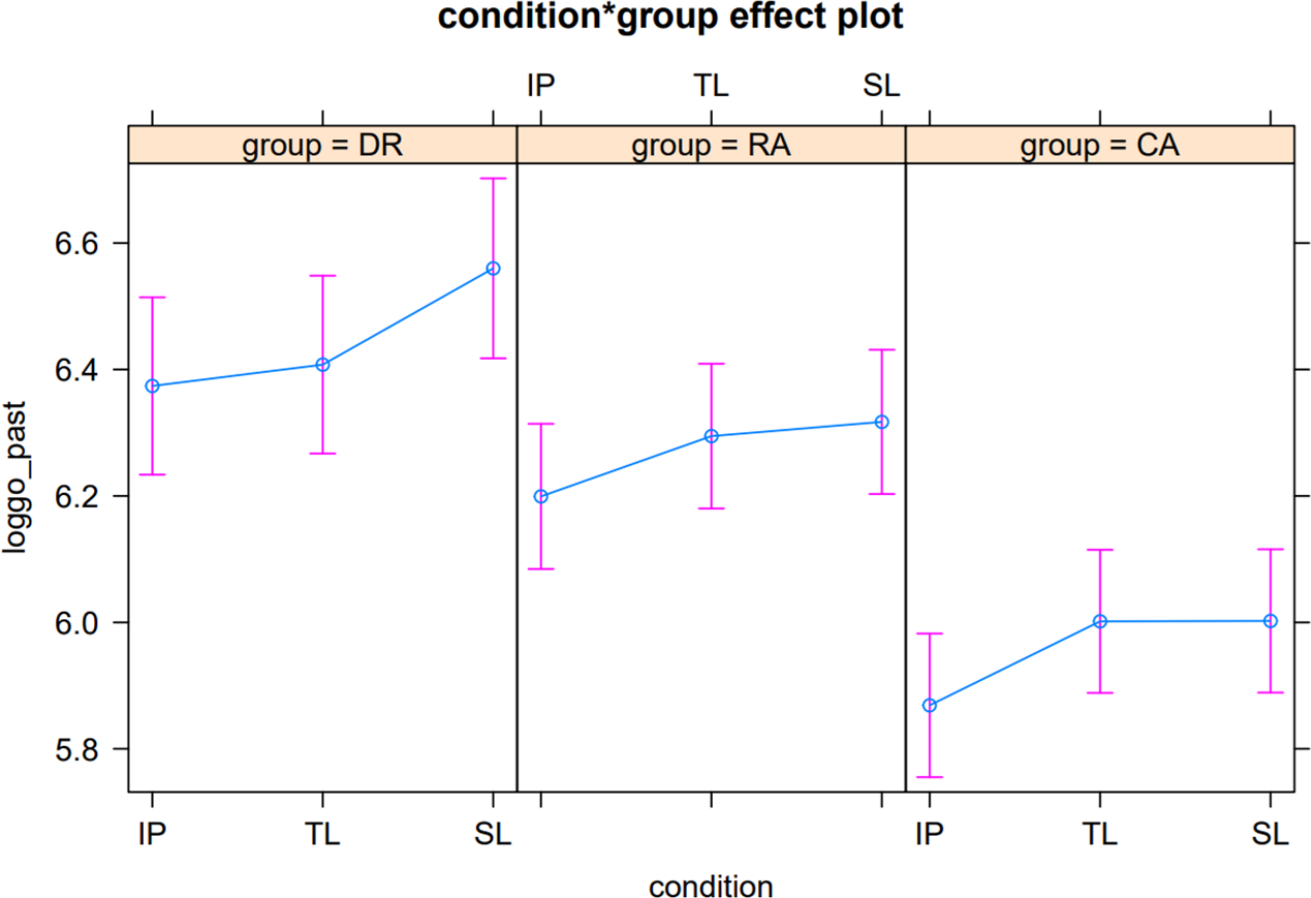
Plots for go‐past time for condition by group interaction.

Dyslexic children and younger reading age matched children required longer go‐past times than the older chronological age matched children. Moreover, dyslexic children required longer go‐past times than the younger reading age matched children. Like gaze duration, there was a main effect of preview condition contrast between the identity condition and the TL condition. When predictions from the model were plotted (see Figure 1) taking the random factors of subjects and items into account, however, the dyslexic readers did not show shorter go‐past times in the TL condition compared with the identity condition. There was also a main effect of condition for the contrast between the TL condition and the SL condition. There was, also a significant interaction demonstrating that dyslexic readers show a greater benefit of TL previews compared with SL previews than the typically developing readers. Simple effects analysis was conducted to explore the TL effect for dyslexic children (see Table 7).

#### 3.4.5 Fixation Count

Dyslexic children and the younger reading age matched children required more fixations than the older chronological age matched children. In addition, dyslexic children required more fixations compared with the younger reading age matched children. There were no significant effects for the preview contrasts as well as no evidence to support any of the interactions (see Table 6).

#### 3.4.6 Landing Position

While dyslexic readers show a numerical trend to support a pattern of earlier landing positions on target words compared with both chronological and reading age matched children, the group effects were non‐significant. Furthermore, there were no effects of preview condition on landing position and the interactions were non‐significant (see Table 6).

## 4 Discussion

The current study investigated parafoveal processing in children with and without dyslexia during silent sentence reading. There were four key findings from the present study: (1) both dyslexic and non‐dyslexic children were able to pre‐process information from the parafoveal word; (2) there was a TL effect found for typically developing readers aged ~10 years of age, such that a transposition of letters resulted in shorter reading times than if they were substituted. This affect was only found in single fixation duration for the younger group of typically developing children ~8 years old; (3) a TL effect was found for dyslexic readers; and (4) dyslexic readers showed a reduced sensitivity to TLs relative to that of typically developing readers in go‐past time. We consider each of these in turn.

As predicted all three groups of child readers did parafoveally pre‐process words, where they showed a preview benefit (i.e., subsequent reading times were shorter when the target was fixated) of the IP condition compared with the TL preview condition. Indicating that orthographic information was encoded from the parafovea by dyslexic and typically developing readers as young as ~8 years of age during sentence reading. The skilled child readers ~10 years old, exhibited a difference in the time course of orthographic pre‐processing, whereby parafoveal encoding was evident in first fixation and single fixation duration. While we provide initial evidence that dyslexic child readers do gain orthographic parafoveal preview benefits during reading this was demonstrated in later measures of reading, that is, gaze duration.

Furthermore, all three reading groups were found to benefit from TL previews more than SL previews (i.e., a TL effect). Here specific comparisons were conducted for reading times following previews containing transposed versus SLs. We found a TL effect for dyslexic readers in single fixation duration, gaze duration and go‐past times. A similar TL effect was found for the older more skilled typically developing readers. The younger group of typically developing child readers showed a TL effect only for single fixation duration, where this group of younger readers were typically disrupted equally following a TL preview as they were following a SL preview. Thus, the data indicate that dyslexic readers make use of parafoveal information during reading, and extract letter identity information independently to letter position information. The pattern of results in later reading measures, that is, gaze duration supports a flexible letter position encoding mechanism, similar to that found in adults, and typically developing children (e.g., Johnson, Perea, and Rayner 2007; Pagán, Blythe, and Liversedge 2016).

Our findings support models of letter position encoding such as Overlap (Gómez, Ratcliff, and Perea 2008), Open Bigram (Grainger, Kiyonaga, and Holcomb 2006), SOLAR (Davis 1999, 2010) and SERIOL (Whitney 2001). Thus, our data are also consistent with previous evidence from isolated word recognition (see Grainger 2008, for a review) and reading (e.g., Johnson, Perea, and Rayner 2007; Kirkby et al. 2022; Pagán, Blythe, and Liversedge 2016). Based on the pen and paper assessments reported in the current study dyslexic children with a mean reading age of ~8 years can be attributed similar lexical parafoveal processing typically associated with more skilled reading.

The pattern of results found in gaze duration shows that dyslexic children demonstrated a typical pattern in parafoveal processing, similar to that found in skilled reading (Johnson, Perea, and Rayner 2007), wherein gaze durations in the identity condition were shorter than those in the TL condition, which in turn were shorter than gaze durations in the SL condition. However, for later measures of reading, that is, go‐past times, dyslexic readers demonstrated a reduced sensitivity for encoding letter position such that go‐past times in the TL condition did not significantly differ to those in the identity condition. Dyslexic readers were significantly less disrupted by the TL previews than both chronological‐age and reading‐age control groups. Specifically, this pattern of results in go‐past time indicates that TL previews activate the base words as effectively as the IP for dyslexic readers, which is consistent with evidence from isolated word recognition (e.g., Perea and Lupker 2003, 2004). The reduced disruption to reading found in go‐past times in the TL condition indicates that dyslexic readers have a lower threshold for what represents the target word in preview compared with typically developing readers. When dyslexic readers were presented with a TL nonword, the position that corresponded to each letter in the sequence was not precisely encoded—increased position uncertainty assumption (Gómez, Ratcliff, and Perea 2008). This is potentially due to less rich and fully specified lexical representations. Subsequent go‐past times in the SL condition were increased compared with those found in the TL and identity conditions for dyslexic readers. We attribute this pattern of effects to a less rich and fully specified lexical representation, which cause a failure to detect the transposition of letters in preview. However, SLs in preview were a sufficient cue to detect a mismatch between the preview and target word and as such this effect is unlikely to be due to foveal load. This pattern of effects was not found for the typically developing children matched for reading‐age and chronological‐age.

Recent evidence has shown parafoveal processing of phonology in dyslexic readers comparable to that of their typically developing peers (Blythe et al. 2020), suggesting phonological recoding for those with dyslexia (i.e., fast, pre‐lexical activation of phonology); together with the current findings concerning parafoveal processing (and Kirkby et al. 2022), parafoveal pre‐processing of orthographic characteristics of the word would also appear to be a skill evident in dyslexic readers.

The results for non‐dyslexic child readers demonstrate that parafoveal preview benefit develops alongside reading skill. For younger, less skilled readers, orthographic parafoveal preview benefits occur in later measures of reading, that is, gaze duration and go past time. As they become more skilled readers, reading becomes more efficient and preview effects begin to occur in early measures of reading, that is, first fixation duration and single fixation duration. This is in line with a developmental increase in the rate of lexical processing (Reichle et al. 2013) and the findings that lexical processing is slower in children compared with adults (e.g., Blythe 2014; Blythe et al. 2006, 2009; Häikiö et al. 2009; Häikiö, Bertram, and Hyönä 2010; Huestegge et al. 2009; Joseph et al. 2009; McConkie et al. 1991; Rayner 1986; Rayner, Ardoin, and Binder 2013; Reichle et al. 2013; Tiffin‐Richards and Schroeder 2015). By the time typically developing child readers reach the age of approximately 11 years old, they demonstrate similar foveal and parafoveal processing abilities to that of adult readers (Blythe and Joseph 2011; Pagán, Blythe, and Liversedge 2016). Indeed, the chronological age matched group of children in the current study showed parafoveal preview benefit for IPs in all measures of reading, and a TL effect in single fixation duration and gaze duration. Thus, non‐dyslexic skilled readers performed similar to those in Pagán, Blythe, and Liversedge (2016) and Tiffin‐Richards and Schroeder's (2015) studies, which provide further confirmation of the development of parafoveal processing.

Regarding dyslexic reading, the current results provide evidence that dyslexic child readers can gain parafoveal preview benefit during silent sentence reading and extends the body of work demonstrating dyslexic adult readers gain parafoveal preview benefits during silent reading (Kirkby et al. 2022), during RAN (Jones, Ashby, and Branigan 2013) and children during RAN (Yan et al. 2013). Furthermore, we found dyslexic children show similar orthographic parafoveal preview benefits in gaze duration as those found for typically developing child readers matched for chronological age, that is to say, they encoded letter identity and letter position information from the parafovea. We know that dyslexic readers tend to read less and have smaller vocabularies (Cunningham and Stanovich 1998) and less well‐specified phonological representations (Snowling 2001), that is, reduced quality lexical representations. We found that dyslexic readers did not require the same level of specificity with respect to letter position within a word in order to parafoveally process the upcoming word as effectively as non‐dyslexic readers. This was demonstrated in go‐past time, where, in comparison to typically developing child readers matched on reading‐age and chronological‐age, dyslexic readers were less disrupted by TLs. Furthermore, typically developing children matched on reading‐age to the dyslexic readers demonstrated similar levels of disruption in the TL condition as they did in the SL condition in later measures of reading, namely, gaze duration and go‐past time.

Both adult dyslexic readers (Kirkby et al. 2022) and children with dyslexia clearly do gain parafoveal preview benefit during reading. However, in contrast to the current findings Kirkby et al. (2022) found that adult dyslexic readers demonstrated the typical disruption in go‐past times when previews contained TLs. Whereas the position that corresponded to each letter in the TL preview was not precisely encoded by children with dyslexia and go‐past times where not significantly increased compared with those following an IP. The reduced sensitivity to orthographic encoding found in children, but not adults with dyslexia (Kirkby et al. 2022), indicates that with time and continued exposure to print, dyslexic readers develop higher‐quality lexical representations, leading to greater sensitivity to orthographic encoding during parafoveal processing.

In the current study we also found group differences in foveal processing between dyslexic and typically developing children match for chronological age; dyslexic readers had longer reading times across, first fixation durations, single fixation durations, gaze durations and go‐past times. We also found typically developing children made fewer first pass fixations on words than dyslexic children. Dyslexic readers' relatively reduced‐quality lexical representations clearly impact lexical identification and slow down the retrieval process of the fixated word.

Arguably the more informative comparisons are differences between dyslexic readers and the children matched for reading age (and hence chronologically, 2–3 years younger than the dyslexic readers). Overall, the current data demonstrated differences between these reading‐age matched groups such that dyslexic readers required longer gaze durations and go‐past times on target words and an increased number of fixations compared with the reading‐age matched children. Within‐word refixations indicate problems the reader has processing the fixated word (Rayner 1998). In this case, compared with children with a matched reading‐age, the dyslexic readers engaged in significantly more within‐word refixations. In addition to the well‐established phonological deficits, evidence suggests that children with dyslexia have impaired speed of processing—where the most established evidence has come from the RAN task (e.g., Jones, Ashby, and Branigan 2013; Yan et al. 2013). The increase in reading times for dyslexic readers compared with reading‐age matched children may also reflect a dyslexia specific processing speed deficit, over and above any effect that measures of reading‐age would predict. However, the current study is not able to disentangle these.

In addition, dyslexic readers had increased regressive fixation durations, compared with that of typically developing child readers matched for reading‐age. Longer regressive fixations indicate more sentence processing errors (Murray and Kennedy 1988; Rayner 1998). In this case, the dyslexic readers engaged in longer regressive fixation durations to re‐read aspects of the text compared with children matched for reading‐age.

In sum, the current experiment provides a detailed account of foveal and parafoveal effects on eye movements for dyslexic children during sentence reading. While we provide evidence that dyslexic readers gain orthographic parafoveal preview benefit during silent sentence reading, we found differences in dyslexic reading across foveal and parafoveal processing compared with a reading‐age matched control group. In line with previous theories of dyslexia (e.g., Stanovich 1988) and findings described in Blythe et al. (2020), our results indicate that dyslexic reading difficulties stem from a fundamental deficit, rather than a developmental delay. Furthermore, we demonstrated differences in the time course of parafoveal processing compared with a chronological‐age matched group of child readers. Critically, we demonstrated less effective orthographic encoding in dyslexic reading in go‐past time compared with both chronological‐age and reading‐age matched groups. Although we cannot make strong conclusions as to the cause of this difference, the data demonstrate a consistent dyslexic‐specific difference in both foveal and parafoveal processing during silent sentence reading in comparison to typically developing readers. Dyslexic‐specific deficits are associated with the development of less rich and fully specified lexical representations and as such children with dyslexia have a reduced sensitivity to orthographic encoding during parafoveal processing. These findings are compatible with the view that orthographic representations of dyslexic children are not yet sufficiently specified.

## Conflicts of Interest

The authors declare no conflicts of interest.

## Data Availability

Data openly available in a public repository that issues datasets with DOIs.
